# Application and prospects of fecal microbiota transplantation in cancer therapy

**DOI:** 10.3389/fcimb.2026.1880573

**Published:** 2026-07-31

**Authors:** Yu-Mei Fan, Yu Li, Hai-Yan Bi, Yao Ding, Linya Lyu, Yan Geng, Wei Fu

**Affiliations:** 1 Department of Gastroenterology, The 925th Hospital of PLA Joint Logistics Support Force, Guiyang, China; 2 Department of Gastroenterology, The 923rd Hospital of PLA Joint Logistics Support Force, Nanning, China; 3 Department of Cardiovascular, The Second People’s Hospital of Kunming, Kunming, China

**Keywords:** cancer therapy, fecal microbiota transplantation, gut microbiota, immunotherapy, gut microecology, precision medicine

## Abstract

As the largest community of commensal microorganisms in the human body, the gut microbiota-through its structural and functional imbalances-participates in the entire process of tumor initiation and progression, and profoundly influences the efficacy and safety of conventional anticancer regimens, including chemotherapy and immunotherapy. Fecal microbiota transplantation (FMT) offers a novel biological strategy for cancer treatment by reestablishing the ecological balance of the host gut microbiota. This review systematically integrates the basic theory, clinical practice, technical optimization, epidemiological features, current challenges, and future directions of FMT in oncology. It elaborates on how FMT modulates the tumor microenvironment via core mechanisms such as immune regulation, metabolic reprogramming, and intestinal barrier repair, and shows potential value in enhancing immunotherapy responses, improving chemotherapy tolerance, and alleviating treatment-related complications across various malignancies, including colorectal cancer, liver cancer, and melanoma. Meanwhile, the article outlines practical difficulties regarding donor screening, formulation standardization, ethical oversight, and long-term safety evaluation of FMT, and looks ahead to the development of precision FMT, synthetic microbial consortia, integration of multi-omics technologies, and combination treatment paradigms. The aim is to provide a comprehensive, systematic, and academically in-depth reference for the translational and basic research of FMT in precision cancer therapy.

## Introduction

**Figure 1:**
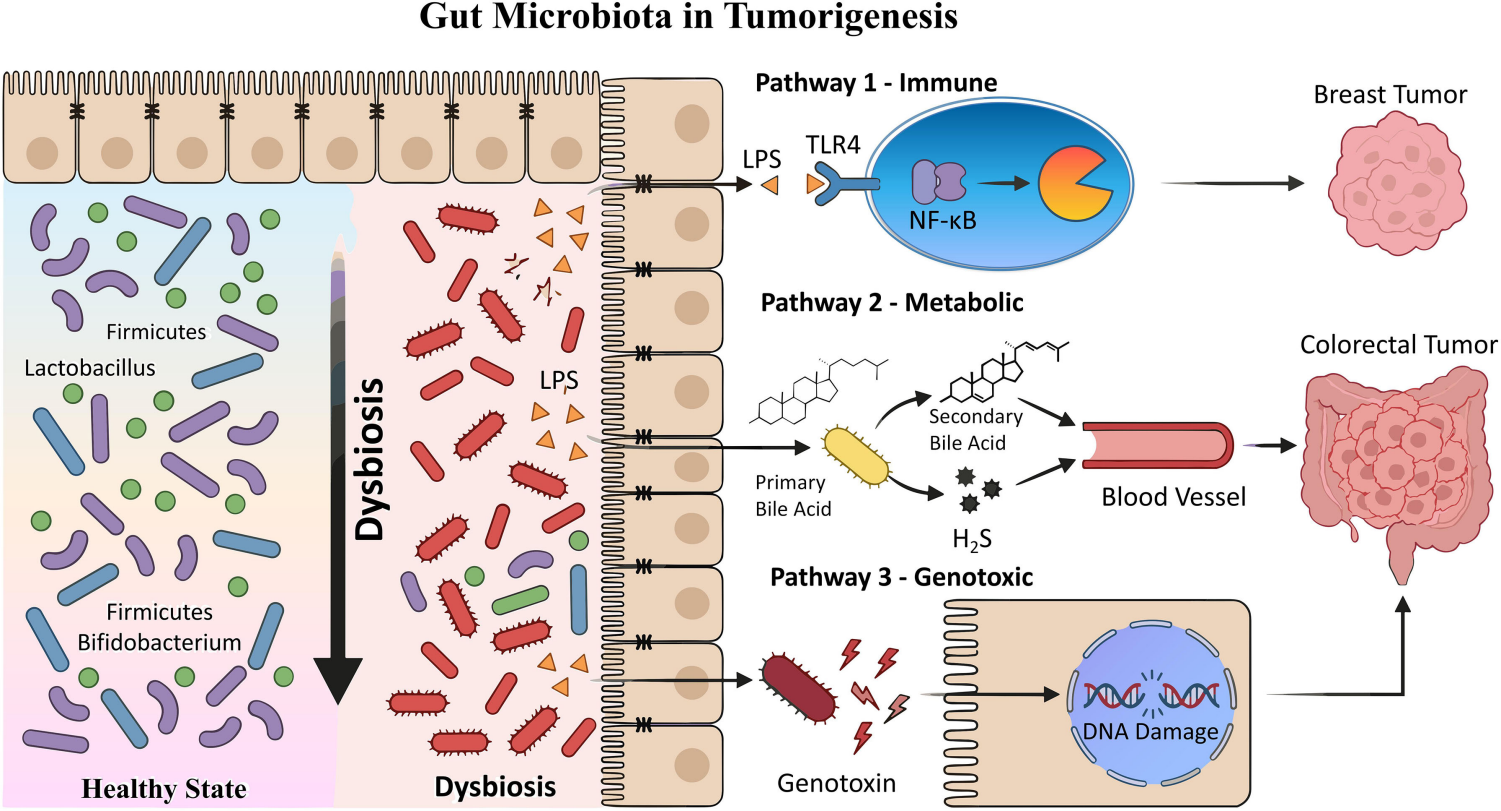
Gut microbiota in tumorigenesis.

The approximately 10^14^ microorganisms colonizing the human intestine together form a complex and stable microecological system that widely participates in core physiological processes, nutrient metabolism, immune development, barrier maintenance, and is a critical component of host homeostasis. In recent years, microbiome research has consistently confirmed that gut dysbiosis is an important driver of tumor initiation and progression, promoting tumorigenesis through multiple pathways such as the accumulation of carcinogenic metabolites, the sustained activation of chronic inflammatory signals, impaired immune surveillance, and the induction of DNA damage (Dong et al., 2019; Ge et al., 2021; Han et al., 2024; Nakatsu et al., 2024)(as shown in **Figure 1**). At the same time, the gut microbiota directly participates in the metabolism of chemotherapeutic drugs, the modulation of immune checkpoint inhibitor (ICI) efficacy, and the development of treatment-related toxicities, making it a key regulatory target that determines the success or failure of cancer therapy (Sadeghloo and Sadeghi, 2025; Shi et al., 2025; Ung and Bonavida, 2025; Xie and Liu, 2024). Recent literature delineates complex microbiome-pancreatic cancer interactions, highlighting microbial control over inflammation, tumor immunity and therapeutic unresponsiveness (Jagwani et al., 2025).

Fecal microbiota transplantation, an intervention that rebuilds the gut microecology, was first successfully used to treat recurrent Clostridium difficile infection. Its core principle is to rapidly correct structural disturbances of the recipient’s microbiota and restore microbial diversity and key metabolic functions by transplanting functional gut microbiota from a healthy donor (Khoruts and Sadowsky, 2016; Li et al., 2016). As research on the tumor microecology deepens, the boundaries of FMT application in oncology continue to expand from initial management of complications gradually toward directly enhancing antitumor immunity, reversing treatment resistance, and improving long-term patient survival, making FMT an important bridge connecting fundamental microbiome research with precision cancer therapy practice. At present, preliminary clinical evidence supports the use of FMT in reversing immunotherapy resistance, alleviating chemotherapy-induced diarrhea, and treating graft-versus-host disease after hematopoietic stem cell transplantation. However, key issues remain, including unclear mechanisms, non-standardized technical protocols, and unverified long-term safety. Based on the latest domestic and international research progress, this article systematically integrates the application value, mechanisms, clinical practices, and technical optimization of FMT in cancer therapy, providing theoretical support for its standardized clinical application and future research directions.

## Biological basis of FMT in regulating tumor initiation and progression

The gut microbiota and the host have a long coevolutionary relationship; the structural stability and functional balance of the microbiota constitute an important defense line against tumorigenesis. When factors such as diet, drugs, or disease cause dysbiosis, the composition and function of the gut microbiota become remodeled, thereby driving tumor development through multiple pathways, including metabolism, inflammation, immunity, and genotoxicity. A dysbiotic microbiota can overproduce potentially carcinogenic metabolites such as secondary bile acids and hydrogen sulfide, while simultaneously reducing the synthesis of protective short-chain fatty acids (e.g.Butyrate), disrupting the integrity of the intestinal epithelial barrier, promoting bacterial translocation, and inducing systemic low-grade inflammation (He et al., 2023; Sivaprakasam et al., 2016). At the signaling pathway level, microbial imbalance leads to increased lipopolysaccharide release, which persistently activates pro-inflammatory and pro-tumor pathways such as TLR4, NF-κB, and Wnt/β-catenin, driving abnormal proliferation and malignant transformation of intestinal epithelial cells (Lee, 2021). On the immune regulation front, a disturbed microbiota reduces the infiltration of effector immune cells (e.g., CD8^+^ T cells, NK cells) and increases the proportion of regulatory T cells and myeloid-derived suppressor cells, creating an immunosuppressive tumor microenvironment that weakens the body’s ability to surveil and eliminate tumor cells (Allegretti et al., 2020; Liu et al., 2021; Macandog et al., 2024). Furthermore, pathogenic bacteria such as Fusobacterium nucleatum and pathogenic Escherichia coli can directly induce host DNA damage via virulence factors, further accelerating the development of gastrointestinal cancers like colorectal cancer (Han et al., 2025). The gut microbiota also interacts with host miRNAs to regulate cell proliferation and apoptosis, playing an important regulatory role in early tumorigenesis.

The core logic of FMT’s antitumor effect lies in reversing tumor-associated dysbiosis by transplanting exogenous, healthy, and functionally intact microbiota, thereby blocking or delaying malignant progression through three levels: immune remodeling, metabolic reprogramming, and intestinal barrier repair. After the donor microbiota enters the recipient’s intestine, it activates the host’s innate and adaptive immune systems, enhances the cytotoxic function of CD8^+^ T cells, promotes dendritic cell maturation and antigen presentation, reduces the abundance of immunosuppressive cells, and converts ‘cold tumors’ into ‘hot tumors’ capable of mounting an immune response, thereby enhancing the therapeutic effect of immune checkpoint inhibitors (Mohseni et al., 2023). In terms of metabolic regulation, FMT restores the balance of key molecules such as short-chain fatty acids, bile acids, and tryptophan metabolites, ameliorates metabolic disturbances in the tumor microenvironment, and suppresses malignant proliferation of tumor cells (Ranhotra. Regarding intestinal barrier protection, the transplanted microbiota upregulates the expression of tight junction proteins (e.g., ZO1, occludin), reduces intestinal permeability, decreases bacterial translocation and diffusion of inflammatory cytokines, and alleviates secondary damage to the intestinal mucosa caused by radiotherapy, chemotherapy, and immunotherapy (Fan et al., 2026; Halsey et al., 2023). Numerous studies indicate that the efficacy of FMT is highly dependent on the intrinsic characteristics of the donor microbiota; microbiota richness, stability, short-chain fatty acid-producing capacity, and immunomodulatory function are key factors determining treatment outcomes. Moreover, the colonization efficiency and clinical response rate of microbiota transplanted via the colonic route are significantly better than those of the upper gastrointestinal route (Haifer et al., 2022).

The bidirectional interaction between the gut microbiota and the immune system is the core biological basis for FMT’s effects. Beneficial bacteria such as Bifidobacterium, Bacteroides fragilis, and Akkermansia muciniphila promote normal immune cell development and maintain a balance between immune tolerance and inflammation; dysbiosis breaks this balance, induces chronic inflammation, and drives tumor progression (Cerf-Bensussan and Gaboriau-Routhiau, 2010; Huang et al., 2024; Maynard et al., 2012). In the tumor immune regulatory network, microbial metabolites regulate T-cell differentiation and function through signaling pathways such as GPR43 and the aryl hydrocarbon receptor, directly affecting the host’s response to immunotherapy. FMT can effectively repair this interactive pathway and restore the body’s antitumor immune surveillance function (Macandog.

## Current clinical status of FMT in cancer therapy

Based on a clear biological rationale, FMT has been explored clinically in various solid tumors and hematologic malignancies, showing considerable potential. In colorectal cancer, FMT significantly reduces the incidence of chemotherapy-induced diarrhea, increases the proportion of peripheral blood CD8^+^ T cells, improves chemotherapy tolerance and immunotherapy response, and prolongs disease-free survival (Han et al., 2024). In liver cancer, FMT via the gut-liver axis reduces endotoxemia, improves liver function and Child-Pugh score, and, combined with sorafenib targeted therapy, further elevates the objective response rate. In melanoma, for patients resistant to immune checkpoint inhibitors, FMT reshapes the gut microbiome and enables some patients to regain response to immunotherapy, with a marked increase in objective response rate and concomitant upregulation of beneficial bacteria such as Lachnospiraceae and Ruminococcaceae (Meng et al., 2024). In other solid tumors, FMT improves nutritional status and quality of life in pancreatic cancer patients, alleviates chemotherapy-induced intestinal injury in breast cancer, and shows preliminary synergistic potential with immunotherapy in lung cancer (Soto-Pantoja et al., 2021).

In hematologic malignancies and hematopoietic stem cell transplantation, FMT also demonstrates important clinical value. Patients after allogeneic hematopoietic stem cell transplantation often develop severe gut dysbiosis, which is closely associated with increased risks of acute graft-versus-host disease (GVHD), infection, and mortality. FMT can significantly reduce the incidence of acute GVHD, improve one-year survival, and decrease intestinal colonization by multidrug-resistant bacteria as well as bloodstream infection risk (Henig et al., 2021). In CAR-T cell therapy, the status of the gut microbiota is closely related to cytokine release syndrome, neurotoxicity, and treatment response. Preliminary explorations suggest that FMT can attenuate systemic hyperinflammation by modulating microbiota balance, potentially optimizing the safety and efficacy of CAR-T therapy (Ciernikova et al., 2021).

Gastrointestinal complications arising from cancer treatment are major factors limiting treatment intensity and patient compliance, and FMT has also shown outstanding efficacy in salvage therapy of such complications. For steroid-refractory immune checkpoint inhibitor-associated colitis, FMT has become an effective salvage option, rapidly alleviating clinical symptoms, repairing mucosal damage, and reestablishing microbiota balance, with clinical remission rates exceeding 80% (Elkrief et al., 2024). At the same time, FMT effectively alleviates chemotherapy-induced mucositis, severe diarrhea, recurrent infections, and other complications, reducing treatment discontinuation rates and ensuring the smooth delivery of anticancer regimens.

Existing clinical data confirm that FMT has a favorable short-term safety profile in cancer patients. Adverse effects are mainly mild gastrointestinal symptoms such as diarrhea, abdominal pain, and nausea, with a low incidence and most resolving spontaneously; no severe infections or transplantation-related serious adverse events have been reported. Long-term follow-up results show that cancer patients receiving FMT have not experienced increased long-term safety issues such as infections, autoimmune diseases, or elevated tumor recurrence risk, providing preliminary reassurance for its long-term use (El-Salhy et al., 2022). In terms of efficacy, the response rate of FMT for reversing immunotherapy resistance is approximately 30–40%, the rate for improving chemotherapy-related toxicity is around 60–70%, and the efficacy rate for treating GVHD is about 75%; moreover, efficacy is positively correlated with the abundance of beneficial bacteria in the donor. **Table 1** summarizes representative clinical trials of fecal microbiota transplantation (FMT) across cancer therapies, reflecting the burgeoning interest in this approach as a promising adjunct to conventional oncology regimens.

**Table 1:** Fecal microbiota transplantation (FMT) in cancer therapy: summary of registered clinical trials.

NCT number	Phase	Cancer type	Intervention	Status	Primary endpoint	Location	Start date
		Melanoma					
NCT03341143	Phase 2	Melanoma	FMT + Pembrolizumab	Completed	ORR	UPMC Hillman Cancer Center, USA	2018-03
NCT04988841	Phase 2	Melanoma	MaaT013 (pooled-donor FMT) + Ipilimumab + Nivolumab	Completed	Safety of 23-week combination	France (multicenter)	2022-01
NCT03772899	Phase 1	Melanoma	FMT + immunotherapy	Active, not recruiting	Safety (FMT + ICI combination)	LRCP/CHUM/JGH, Canada	2019-03
NCT03353402	Phase 1	Melanoma	FMT	Unknown	FMT-related AEs; Microbiome engraftment	Sheba Medical Center, Israel	2017-11
NCT06030037	Phase 2	Melanoma	Healthy donor FMT + Pembrolizumab + Lenvatinib	Withdrawn	ORR	UPMC, USA	—
Non-small cell lung cancer							
NCT05669846	Phase 2	NSCLC	Healthy donor FMT + Pembrolizumab	Recruiting	ORR (RECIST 1.1)	UPMC Hillman Cancer Center, USA	2025-01
NCT06403111	Phase 2	NSCLC	FMT + chemotherapy + immunotherapy	Recruiting	12-month PFS rate	Changzhou No.2 People’s Hospital, China	2024-06
NCT07432984	Phase 2	NSCLC	FMT + Tislelizumab	Not yet recruiting	Safety (SAE/AE)	Seoul,KAorea	2026-04
NCT05008861	Phase 1	NSCLC	Capsulized FMT + anti-PD-1/PD-L1 + platinum-based chemotherapy	Unknown	FMT- and ICI-related AE incidence	China	2021-09
Lung cancer							
NCT04924374	N/A	Lung Cancer	Microbiota transplant + anti-PD1	Completed	Safety	Hospital Ramón y Cajal, Spain	2021-04
NCT05502913	Phase 2	Lung Cancer	Antibiotics + FMT + ICI	Recruiting	PFS	Soroka/Rabin, Israel	2023-09
Gastrointestinal, colorectal & pancreatic cancer							
NCT04130763	Phase 1	GI Cancer	FMT capsule + anti-PD-1	Completed	ORR; AEs	Beijing Cancer Hospital, China	2019-12
NCT04729322	Phase 2	CRC	FMT/FMT capsule + Pembrolizumab/Nivolumab + antibiotic pretreatment	Active, not recruiting	ORR	USA	2021-02
NCT06393400	Phase 1	Pancreatic Cancer	FMT + PEG + Gemcitabine + nab-Paclitaxel	Recruiting	AEs	Canada	2025-01
Hepatocellular carcinoma (HCC)							
NCT05750030	Phase 2	HCC	FMT + Atezolizumab + Bevacizumab	Completed	Safety (TRAE incidence)	Medical University of Vienna, Austria	2023-05
NCT07463248	Phase 2	HCC	PULSAR + FMT + Tislelizumab + TKI	Not yet recruiting	PFS	China	2026-03
Renal cell carcinoma (RCC)							
NCT04163289	Phase 1	RCC	FMT + Ipilimumab/Nivolumab	Active, not recruiting	ICI-related colitis incidence	LRCP, Canada	2020-01
		Nasopharyngeal Carcinoma (NPC)					
NCT06486220	Phase 3	NPC	Gut microbiota lyophilized powder capsules + PD-1 antibody	Not yet recruiting	PFS	Sun Yat-sen University Cancer Center, China	2024-07
Breast cancer							
NCT07292142	Phase 1/2	Breast Cancer	FMT + chemotherapy/immunotherapy (neoadjuvant)	Not yet recruiting	Safety; Feasibility	Canada	2026-02
Mesothelioma							
NCT04056026	Early Phase 1	Mesothelioma	FMT + Pembrolizumab	Completed	PFS	USA	2018-09
		Lymphoma & Hematologic Malignancies					
NCT02928523	Phase 1/2	AML	Autologous FMT	Completed	Microbiome dysbiosis prevention	France	—
NCT06218602	Phase 2	Lymphoma	FMT + chemotherapy + CAR-T (Axicabtagene Ciloleucel)	Recruiting	Safety (AEs)	USA	2024-02
NCT03720392	Phase 2	Hematologic malignancies	FMT vs Placebo	Terminated	Gut microbiome diversity recovery	USA	—
Pan-Solid tumors & ICI-related toxicity management							
NCT05273255	N/A	Solid tumors	FMT	Completed	Gut microbiome community change	Swiss	2022-03
NCT04264975	N/A	Solid tumors	FMT	Recruiting	ORR	Asan Medical Center, Korea	2018-06
Melanoma							
NCT04521075	Phase 1/2	Melanoma/MSI-H/NSCLC	FMT capsules + Nivolumab	Unknown	FMT-related AEs; ORR	Sheba Medical Center, Israel	2020-11
NCT05286294	Phase 2	Melanoma/HNSCC/RCC/NSCLC	FMT (from ICI-responder donors)	Active, not recruiting	Safety; Tumor response	Oslo University Hospital, Norway	2022-06
NCT04038619	Phase 1	GU/Melanoma/Lung	FMT + Loperamide	Recruiting	FMT-related AEs; Clinical remission of colitis	MD Anderson, USA	2021-02
NCT03819296	Phase 1	Melanoma/NSCLC/GU	FMT ± Infliximab/Vedolizumab	Recruiting	Stool microbiome change; FMT AEs	MD Anderson, USA	2021-02

FMT, Fecal Microbiota Transplantation; ICI, Immune Checkpoint Inhibitor; ORR, Objective Response Rate; PFS, Progression-Free Survival; AE, Adverse Event; TRAE, Treatment-Related Adverse Event; HCC, Hepatocellular Carcinoma; RCC, Renal Cell Carcinoma; NSCLC, Non-Small Cell Lung Cancer; CRC, Colorectal Cancer; NPC, Nasopharyngeal Carcinoma; HNSCC, Head and Neck Squamous Cell Carcinoma; GU, Genitourinary; AML, Acute Myeloid Leukemia; CAR-T, Chimeric Antigen Receptor T-cell.

## Technical optimization and precision treatment strategies for FMT

**Figure 2:**
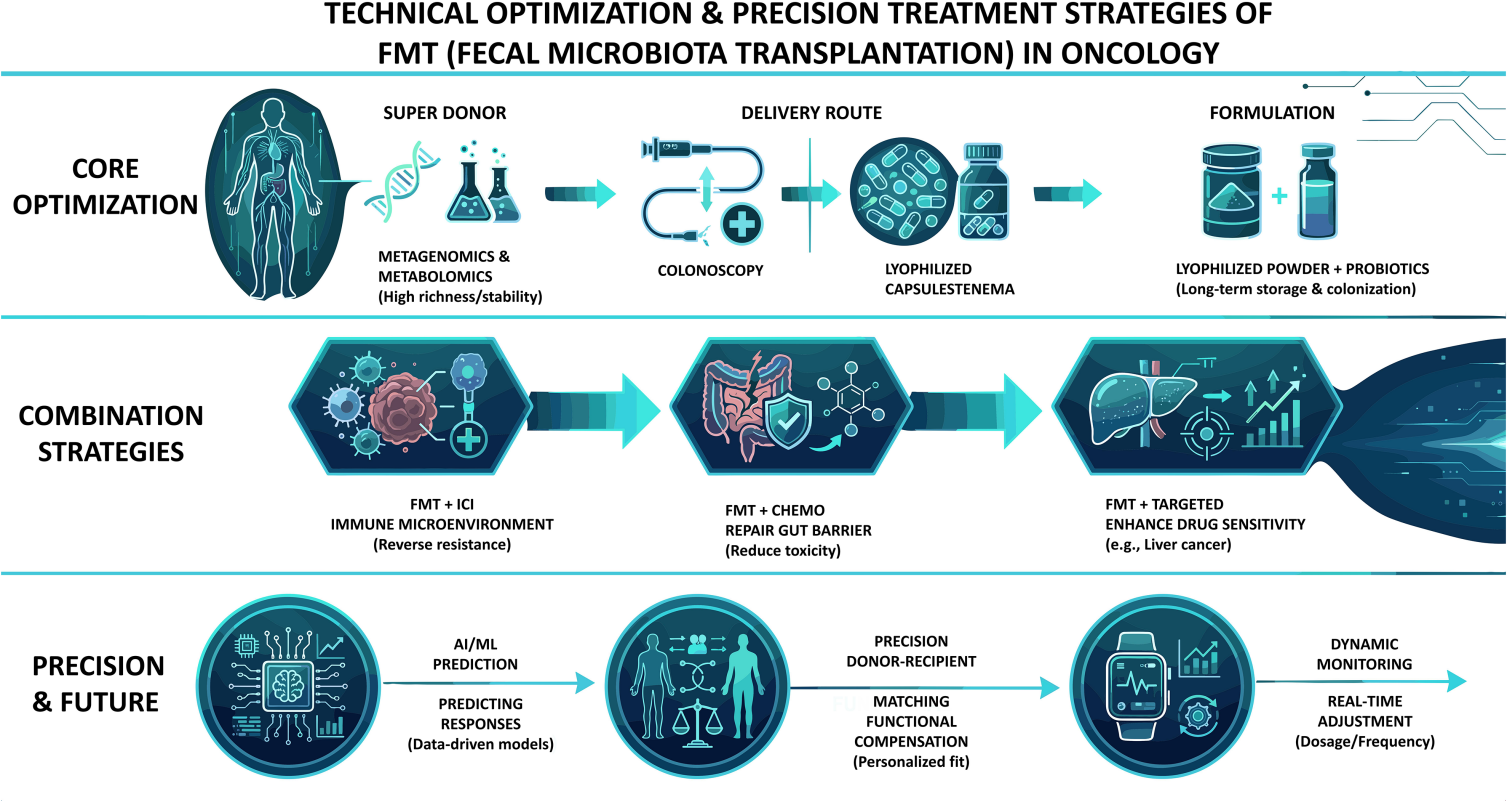
Technical optimization and precision treatment strategies for FMT.

With increasing clinical demand, FMT technology is rapidly evolving from empirical practice toward standardization and precision. The core optimization areas focus on donor selection, transplantation routes, formulation preparation, and combination strategies. Traditional donor screening relies solely on health status assessment, which hardly guarantees the functional effectiveness of the microbiota. New-generation screening systems incorporating metagenomics, metabolomics, and immune characterization enable precise identification of “super donors” with high richness, high stability, and specific antitumor functions, significantly improving treatment response rates. Regarding transplantation routes, although colonoscopic administration is effective, it is highly invasive; non-invasive methods such as oral lyophilized capsules and rectal enemas are gradually becoming more clinically applicable due to better compliance and comparable efficacy (Sipos et al., 2024). Formulation standardization is key to large-scale application of FMT; lyophilized preparations allow long-term stable storage, solving the storage and transportation problems of fresh fecal samples. Combining with probiotics or prebiotics can further improve donor microbiota colonization efficiency and enhance therapeutic outcomes (Soto-Pantoja et al., 2021) (**Figure 2**).

Combination therapy is an important direction to enhance the clinical value of FMT, achieving complementary advantages with existing anticancer modalities. FMT combined with immune checkpoint inhibitors remodels the immune microenvironment, reverses treatment resistance, and significantly improves objective response rate; combined with chemotherapy, it repairs the intestinal barrier, reduces mucosal injury, lowers treatment toxicity, and improves chemotherapy completion rate and dose intensity; combined with targeted therapy, it modulates host metabolic pathways and enhances drug sensitivity, with synergistic benefits already shown in liver cancer.

Personalization and precision are the core trends for future FMT development. The key lies in achieving precise donor-recipient matching based on the patient’s baseline microbiota profile, tumor type, treatment history, and immune status. By analyzing the patient’s microbiota deficiencies through metagenomic sequencing and selecting donor microbiota with corresponding functional compensation, colonization rates and clinical efficacy can be significantly improved. Machine learning models can predict a patient’s response to FMT in advance, identify populations most likely to benefit, and optimize treatment timing by performing FMT intervention before immunotherapy often yields better outcomes. Dynamic monitoring of changes in microbiota structure and immune parameters during treatment allows real-time adjustments of dosage, frequency, and combination regimens, achieving precise regulation and further elevating overall response rates.

## Epidemiological characteristics and population differences of FMT

Epidemiological evidence shows that gut dysbiosis is closely associated with an increased risk of various cancers; individuals with severe microbiota disruption have a 2- to 3-fold higher risk of colorectal cancer, and risks of liver cancer, breast cancer, and other tumors are also significantly elevated. A high-fat diet, long-term antibiotic use, and low dietary fiber intake are important external factors that induce dysbiosis and increase cancer risk, further supporting the potential value of microbiota intervention in cancer prevention.

Improvement in survival outcomes among cancer patients receiving FMT has been supported by multiple clinical studies. In melanoma patients, FMT combined with immunotherapy significantly increases 3-year survival; in colorectal cancer patients, FMT improves long-term survival; in hematopoietic stem cell transplantation patients, FMT markedly raises one-year survival. The core mechanisms include enhanced antitumor immunity, repair of the intestinal barrier, improved metabolic and nutritional status, and reduced infection and complication risks (Henig et al., 2021).

The clinical efficacy of FMT varies significantly across different populations, a feature that provides a basis for selecting optimal candidates. Younger age, female sex, and coexisting inflammatory bowel disease are associated with better FMT responses in cancer patients; immunocompromised status, long-term broad-spectrum antibiotic use, and severely impaired gut microbiota diversity predict weaker responses. Different tumor types also show differential responses to FMT: tumors closely linked to the microbiota, such as melanoma and colorectal cancer, respond more robustly, whereas pancreatic cancer shows relatively modest responses. This suggests that clinical application should appropriately select indications based on tumor biological characteristics.

## Challenges and controversies facing FMT

Although FMT holds great promise in cancer therapy, its clinical translation still faces a number of practical challenges, including ethical and regulatory oversight, safety management, definition of indications, and standardization. In terms of ethics and legal regulation, FMT involves issues of donor privacy protection, fully informed consent of recipients, and commercialization norms. Globally, there is no unified regulatory standard yet; different countries and regions have widely varying requirements for donor screening, product management, and clinical application qualifications, creating obstacles for multi-center research and standardized promotion (Mikail et al., 2020).

Long-term safety and potential risks must be carefully addressed in clinical application. Although short-term safety is good, the long-term effects of FMT on host immunity, metabolism, and endocrinology remain unclear. Potential risks include pathogen transmission, induction of autoimmune diseases, and tumor cell dissemination. Although the incidence is low, strict donor screening systems, product quality control systems, and long-term follow-up monitoring systems need to be established to minimize risks.

The definition of indications and contraindications still requires more evidence. Currently, the well-recognized applications of FMT in oncology mainly include immune checkpoint inhibitor resistance, severe chemotherapy-induced toxicity, GVHD after hematopoietic stem cell transplantation, and refractory immune-related colitis. The value of FMT in cancer prevention, adjuvant therapy after surgery, and advanced palliative care is still being explored. Contraindications mainly include severe immunodeficiency, intestinal obstruction or perforation, active systemic infection, and allergy to donor microbiota. As research deepens, the scope of indications will continue to expand, and the contraindication framework will become more refined (Henig et al., 2021).

Insufficient understanding of basic mechanisms and a lack of standardized systems are core bottlenecks limiting FMT development. At present, the key functional strains, core metabolic pathways, and critical immune targets responsible for FMT’s effects have not been fully elucidated; the process remains largely a ‘black box.’ Donor selection criteria, formulation preparation protocols, administration regimens, and efficacy evaluation systems all lack uniform standards, making it difficult to compare results across studies and to generate high-level evidence-based medical recommendations.

## Summary and outlook

As a representative approach for modulating the tumor microenvironment, fecal microbiota transplantation exerts its effects through multiple mechanisms, including immune remodeling, metabolic restoration, and intestinal barrier protection. It has demonstrated important clinical value in enhancing immunotherapy efficacy, reducing treatment-related toxicity, and improving patient survival outcomes, and has undergone initial translation from basic research to clinical exploration. At present, FMT still faces challenges regarding donor screening standardization, formulation normalization, long-term safety validation, precision matching strategies, and ethical regulatory frameworks. However, with the rapid integration of microbiome research, synthetic biology, artificial intelligence, and multi-omics technologies, FMT is evolving rapidly from empirical transplantation toward precision, personalization, and standardization.

In the future, FMT development will focus on four directions: first, deepening mechanistic research to decipher the microbiota-metabolite-immune-tumor microenvironment regulatory network and identify core functional strains and key effector molecules; second, establishing standardized technical systems, including unified donor screening, formulation preparation, quality control, and clinical operation protocols; third, developing next-generation precision intervention products such as synthetic microbial consortia, customized postbiotic formulations, and targeted delivery systems to improve efficacy and safety; fourth, advancing high-quality clinical research with multi-center, large-sample, randomized controlled trials to define the optimal indications, combination regimens, and treatment timing for FMT in different cancers, leading to authoritative guidelines and consensus.

In summary, fecal microbiota transplantation, as an innovative strategy linking the microbiome and precision cancer therapy, holds substantial translational potential. With continued research and technical refinement, FMT is expected to become a safe, effective, and standardized component of comprehensive cancer treatment, offering new therapeutic options for cancer patients.
